# Edge-dislocation-induced ultrahigh elevated-temperature strength of HfMoNbTaW refractory high-entropy alloys

**DOI:** 10.1080/14686996.2022.2129444

**Published:** 2022-10-17

**Authors:** Ko-Kai Tseng, Hao-Hsuan Huang, Woei-Ren Wang, Jien-Wei Yeh, Che-Wei Tsai

**Affiliations:** a Department of Materials Science and Engineering, National Tsing Hua University, Hsinchu, Taiwan, ROC; b High Entropy Materials Center, National Tsing Hua University, Hsinchu, Taiwan, ROC; c Department of Additive Manufacturing Materials & Applications, Division of Metallic Materials Research, Material and Chemical Research Laboratories, Industrial Technology Research Institute, Tainan, Taiwan, ROC

**Keywords:** High-entropy alloys, refractory high-entropy alloys, solid-solution strengthening, ultrahigh elevated-temperature strength, dislocations, slip mode

## Abstract

Over 150 refractory high-entropy alloys (RHEAs) have been proposed in the last decade. Early alloys such as MoNbTaW and MoNbTaVW still show an unparalleled yield strength of approximately 400 MPa at 1600°C. However, RHEAs with even elevated high-temperature strength are necessary in aerospace vehicles and nuclear reactors to cope with advanced technology in the future. Here, solid-solution strengthening calculation and melting point prediction are combined to design single-phase RHEA for attaining ultrahigh strength at 1600°C. The results show that Hf_0.5_MoNbTaW and HfMoNbTaW alloys after fully homogeneous treatment at 2100°C for 2 h reveal a homogenous body-centered cubic phase. HfMoNbTaW alloy exhibits a yield strength of 571 MPa at 1600°C, much higher than that of MoNbTaVW (477 MPa). It is found that a plateau of strength occurs from 800°C to 1200°C, which is important for raising the strength level of RHEAs at high temperatures. This strengthening mechanism is explained with the change of deformation mode from screw to edge dislocations, which contributes an edge-dislocation-induced strength. A similar alloy design strategy could be applied to develop more RHEAs with an ultrahigh strength level.

## Graphical abstract

**Figure:**
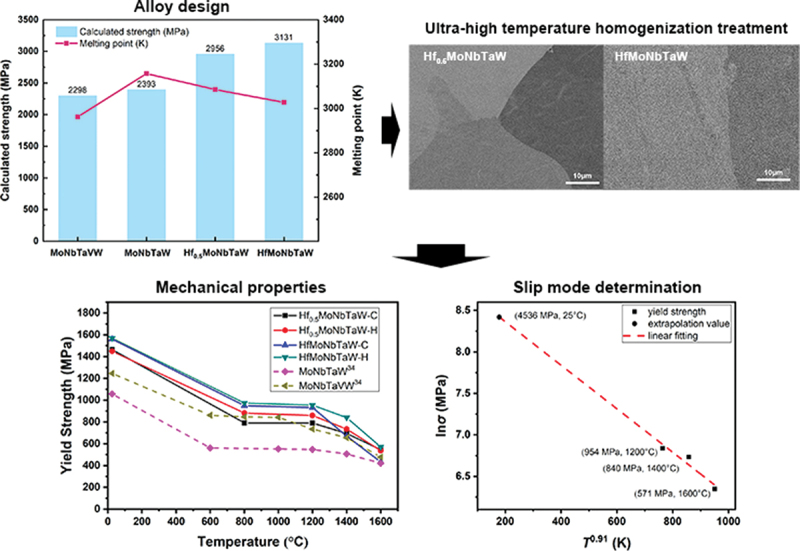

## 1. Introduction

Following the first publications in 2004, the concept of high-entropy alloys (HEAs) has been exploited in alloys design to develop new alloys for academic research and industrial applications [1,2]. Furthermore, various novel design methodologies have been proposed to widen the pool of potential compositions and applications [3,4]. Diffusion theory [5], dislocation theory [6–9], and mechanical properties [10,11] have been used to describe the deformation mechanisms of HEAs at elevated temperatures. However, the trade-off between strength and ductility or a win-win for both is always the goal to pursue, by which high accommodation of active slip planes is proposed to be the key strategy to attain the highest combination of strength and ductility [12]. It has been reported that various alloys have very outstanding mechanical properties [13–17]. Special categories of HEAs, such as refractory high-entropy alloys (RHEAs) [18,19], eutectic HEAs [20], high-entropy superalloys [21], and light-weight HEAs [22,23], also have their unique attractions in mechanical properties and hence the potential for various applications in energy, aerospace, and other industries.

After the first development of RHEAs in 2010, over 150 kinds of compositions have been reported, with their potential for use in high-temperature applications such as gas turbine engines and rocket nozzles. Various studies focused on ductility enhancement [24,25], better oxidation resistance [26], and nuclear reactor material applications [27,28]. According to a review by Senkov et al. [19], the only alloys to pass high-temperature compression tests at 1600°C were MoNbTaW and MoNbTaVW. The main reason for the difficulty to perform compression tests at extremely high temperatures is the lack of suitable material as a jig material that provides high strength at extremely high temperatures. Recently, Wang et al. strengthened the grain boundary of an MoNbTaW-refractory high-entropy alloy through doping with B or C in trace amount, which simultaneously increases room-temperature strength and toughness [29]. This study provides a good strategy to increase the ductility of RHEAs by doping suitable elements.

Several theoretical models of solid-solution strengthening have been proposed to explain how different constituent elements affect room-temperature or elevated-temperature strength [9,30–33]. We previously designed a RHEA that exhibited higher elevated-temperature strength and discussed the effect of the constituent elements on equiatomic HfMoNbTaTiZr alloys by using the subtraction method [33]. For higher room-temperature strength, one should add elements such as Mo, which significantly interact with the other elements. For higher elevated-temperature strength, one should add the elements that possess a high melting point such as Mo, Nb, or Ta. For higher ductility, one should add more Nb. With Ti or Zr addition, both the elevated-temperature strength and the density decrease.

In this study, Hf_0.5_MoNbTaW and HfMoNbTaW RHEAs were designed for extremely high elevated-temperature strength by using the above guidelines: Ti and Zr of HfMoNbTaTiZr were replaced by W to increase the melting point and Young’s modulus. The proposed alloys will be compared with standard MoNbTaVW that contains vanadium instead of hafnium. Moreover, homogenization treatments at a temperature of 2100°C for 2 h, followed by air cooling, were conducted in an ultrahigh-temperature furnace in order to get the fully homogeneous state. Their microstructures and mechanical properties were investigated using scanning electron microscopy (SEM), energy-dispersive spectroscopy (EDS), X-ray diffraction (XRD), and hardness and compression testing. The phase, temperature-dependent strength, and the ultrahigh elevated-temperature strength are discussed with the solution-strengthening mechanism, especially focusing on the contribution of edge-dislocation-induced strength.

## 2. Methods

### 2.1 Materials

All the alloys in this study were manufactured using a vacuum arc melting furnace with a water-cooled copper mold. The purity of pure elements was 99.9 wt%. The raw material was melted in sequence from a high melting point to a low melting point. For example, among Hf, Mo, Nb, Ta, and W, Ta and W were melted first followed by Mo, Nb, and Hf, in sequence. Homogenization treatment was conducted in an ultrahigh-temperature furnace ST-BI01. The furnace was heated using a graphite heater and controlled by using an infrared thermometer. After repeated creation of a vacuum of 3 × 10^−2^ Torr and purge with Ar three times, the sample was heated up to 2100°C at a heating rate of 10°C/min. But the furnace was vented by passing 150-sccm Ar flow and kept at a positive pressure higher than 1 atm when the temperature was raised above 1400°C. According to equation PV = nRT, it is difficult to maintain the vacuum condition above 1400°C. Furthermore, even little amount of oxygen would cause severe oxidation at 2100°C. By keeping Ar gas flowing out of the chamber, the oxygen in the atmosphere would not get into it. After homogenization treatment at 2100°C for 2 h, the sample was moved down to the cooling chamber and subjected to an Ar flow cooling.

### 2.2 Alloy analysis

The microstructures and compositions of the alloys were analysed using an SEM (JEOL-IT100) equipped with EDS capability. An XRD Bruker D2 PHASER was used to check the crystal structure with a scan rate of 8°/min. Hardness was measured by using a Vickers hardness tester (Matsuzawa Seiki MV-1) under 5 kgf. The room-temperature compression tests were conducted with an Instron 4468 universal testing machine, and the high-temperature compression tests were performed on a Gleeble-3500 thermal-mechanical simulator with a strain rate of 10^−3^ 1/s for all compression tests. The cylindrical samples used for compression testing were 5 mm in diameter and 8 mm in height, while the samples used for compression testing at 1600°C were 3.6 mm in diameter and 6 mm in height. Shear modulus was measured via nanoindentation by using a Hysitron TI-980 Triboindenter.

## 3. Results

### 3.1 Microstructure and mechanical properties

**Figure 1.**
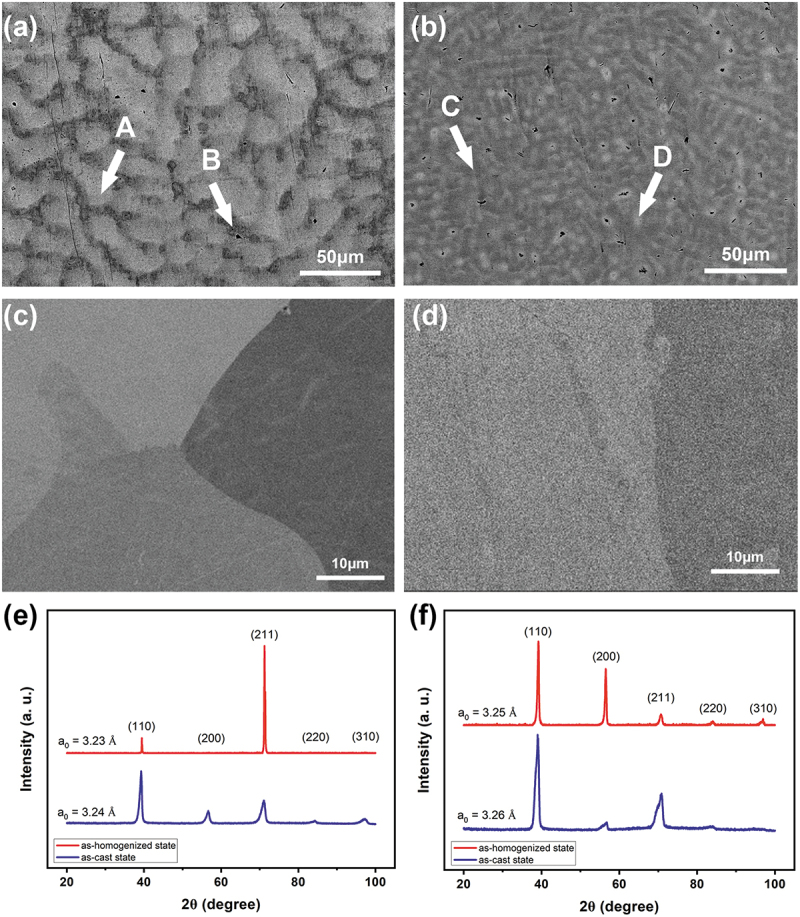
SEM backscatter electron images of (a) Hf_0.5_MoNbTaW and (b) HfMoNbTaW in the as-cast state and (c) Hf_0.5_MoNbTaW and (d) HfMoNbTaW in the as-homogenized state; XRD patterns of (e) Hf_0.5_MoNbTaW and (f) HfMoNbTaW in both states.

Figure 1 shows the microstructure analyses with backscattering electron images and X-ray diffraction patterns of Hf_0.5_MoNbTaW and HfMoNbTaW in both as-cast and as-homogenized states. The compositions of the alloys in both states are shown in Table 1. The as-cast structure is dendritic in which the dendritic region (A) is rich in higher-melting point element, W and Ta, and the interdendric region (B) is rich in the lowest-melting point element, Hf. This is reasonable since higher melting point elements tend to crystallize first from the melt during solidification. It can be noted that the interdendrite of HfMoNbTaW also has a higher Hf content than Hf_0.5_MoNbTaW.

**Table 1.** Compositions of Hf_0.5_MoNbTaW and HfMoNbTaW in the as-cast state (at%).

	Elements	Hf	Mo	Nb	Ta	W
Hf_0.5_MoNbTaW	Nominal composition	11.2	22.2	22.2	22.2	22.2
	Dendrite (A phase)	7.2	22.7	21.4	24.0	24.7
	Interdendrite (B phase)	33.8	22.4	24.4	11.6	7.8
	As-homogenized state	11.6	21.9	22.4	21.7	22.4
HfMoNbTaW	Nominal composition	20.0	20.0	20.0	20.0	20.0
	Dendrite (C phase)	15.3	22.8	22.3	21.4	18.2
	Interdendrite (D phase)	46.0	18.1	18.5	9.3	8.1
	As-homogenized state	20.3	20.6	19.9	19.3	19.9

**Figure 2.**
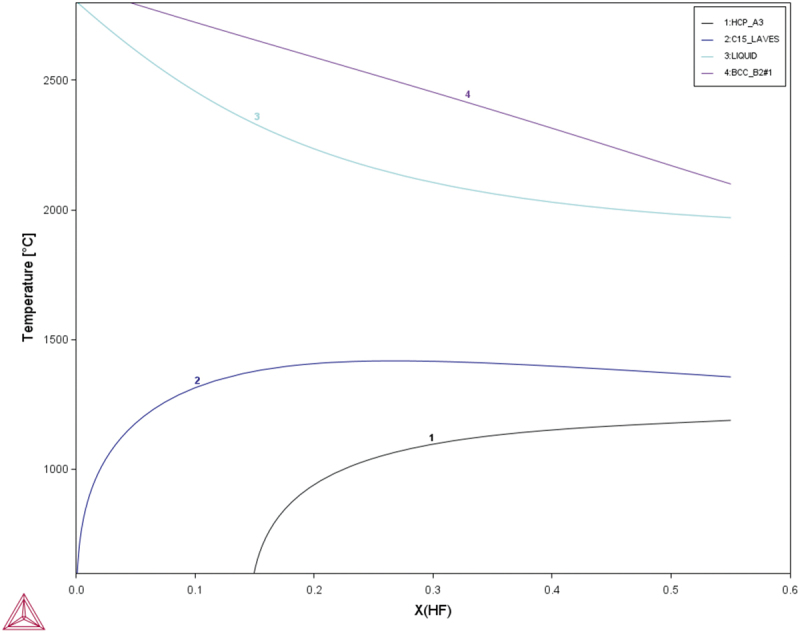
CALPHAD calculation result of different atomic fractions of Hf added to the MoNbTaW system using the TCHEA4 thermodynamic database supplied by ThermoCalc.

According to the previous study [34], MoNbTaW is single phase with a bcc structure. Hf is a Laves phase former. According to Figure 2, when Hf is added to the MoNbTaW system, the bcc phase forms first during solidification, and the remaining becomes the Laves phase. As a result, both the melting point difference and Laves phase formation indicate that the interdendritic phase would be rich of Hf.

**Figure 3.**
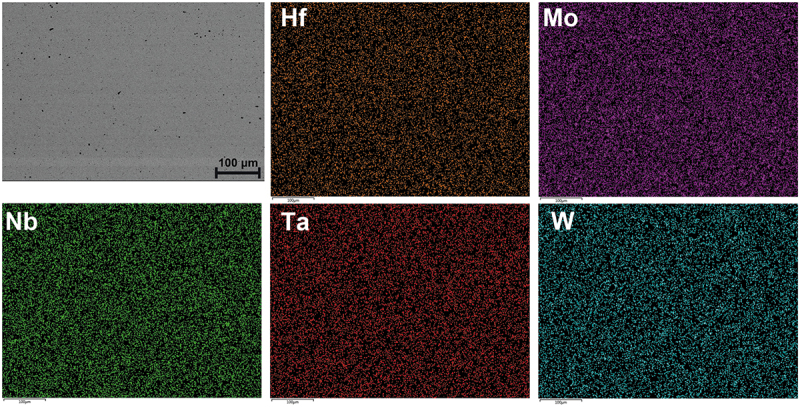
EDS mapping results of as-homogenized HfMoNbTaW alloy.

As the severe partition of Hf would cause problems on mechanical properties, especially at high temperature, homogenization treatment must be performed. Heat treatment at 2100°C for 2 h followed by air cooling indeed ensures complete homogenization and no precipitation during cooling as shown in Figures 1(c,d). The EDS mapping results are also shown in Figure 3. From Figures 1(e,f), the lattice constants of Hf_0.5_MoNbTaW and HfMoNbTaW in the as-cast state are 3.24 and 3.26 Å, respectively, whereas the lattice constants of Hf_0.5_MoNbTaW and HfMoNbTaW in the as-homogenized state are 3.23 and 3.25 Å, respectively. It is apparent that the peak broadening in the as-cast state is due to the composition variation between dendrite and interdendrite regions. The peak of the as-cast state could be decoupled because of the dendritic structure. However, the two decoupled peaks are very close and merge into one peak, which is hard to distinguish.

The hardness values of Hf_0.5_MoNbTaW and HfMoNbTaW in the as-cast state were 543 ± 10 and 550 ± 9 HV, respectively. After homogenization, the hardness values of Hf_0.5_MoNbTaW and HfMoNbTaW were 540 ± 5 and 571 ± 8 HV, respectively. The hardness of the as-homogenized state was almost unchanged compared to that of the as-cast state for Hf_0.5_MoNbTaW, whereas that of HfMoNbTaW increases from 550 HV to 571 HV. This is reasonable as HfMoNbTaW has a larger segregation of Hf in the interdendritic region, and thus, the homogenization process let a larger amount of Hf dissolve into the matrix, which causes a larger solid-solution strengthening effect.

**Figure 4.**
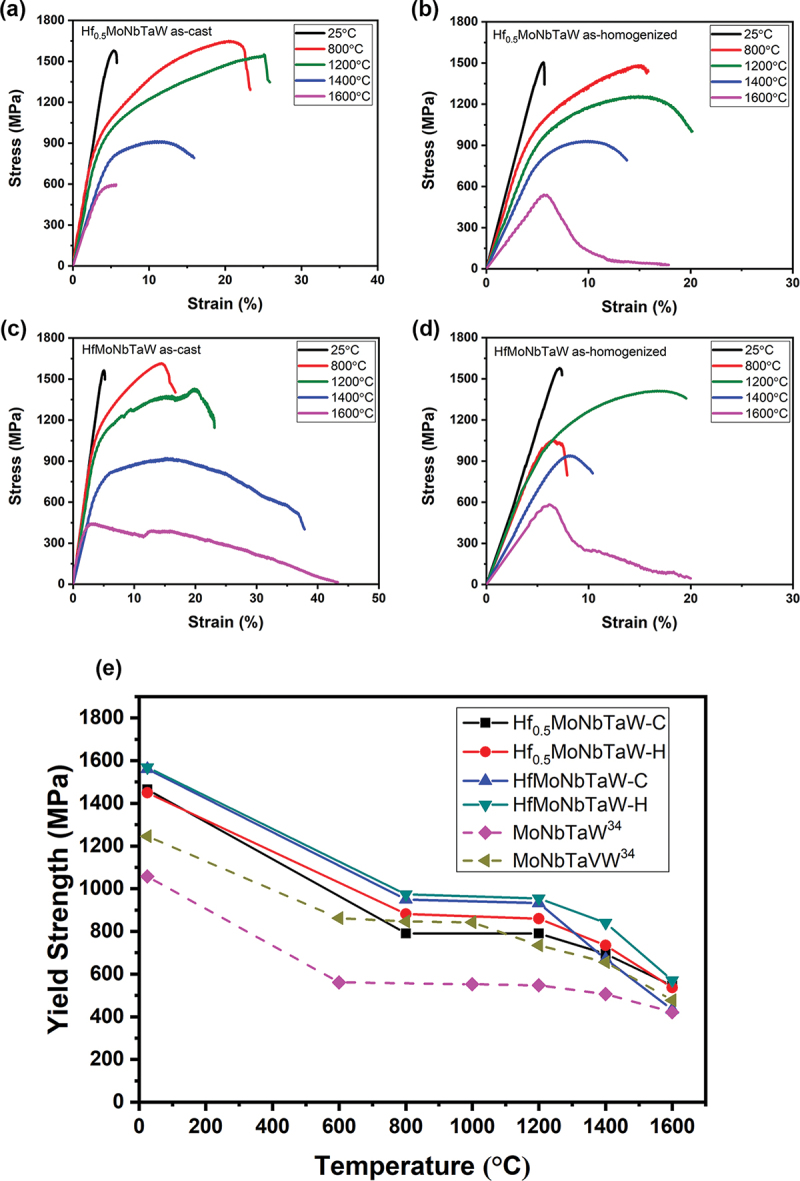
The compression flow stress curves of Hf_0.5_MoNbTaW (a) in the as-cast state and (b) in the as-homogenized state; the flow stress curves of HfMoNbTaW (c) in the as-cast state and (d) in the as-homogenized state; and (e) the temperature dependence of yield strengths of Hf_0.5_MoNbTaW-C and HfMoNbTaW-C in the as-cast state, Hf_0.5_MoNbTaW-H and HfMoNbTaW-H in the as-homogenized state, and MoNbTaW [34] and MoNbTaVW [34].

The flow stress curves of the elevated-temperature compression tests from ambient temperature to 1600°C for Hf_0.5_MoNbTaW and HfMoNbTaW in the as-cast and as-homogenized states are shown in Figure 4. The yield strengths at elevated temperatures are summarized in Table 2. However, the hardness values of Hf_0.5_MoNbTaW and HfMoNbTaW in the as-cast state were 543 ± 10 (5321 MPa) and 550 ± 9 HV (5390 MPa), respectively. By estimating the ultimate strengths of the alloys using the empirical relation that hardness is equal to three times the ultimate strength, in units of MPa [35], it could be noted that the samples were fractured below predicted strengths from their hardness values. In addition, the strength of HfMoNbTaW, 1561 MPa, is not only higher than that of Hf_0.5_MoNbTaW in the as-homogenized state but also much higher than that, 1058 MPa, of MoNbTaW [34]. This indicates that adding Hf into the MoNbTaW can increase yield strength by 47.5% due to the strong solution-hardening effect.

**Table 2.** The yield strengths (MPa) at different temperatures for Hf_0.5_MoNbTaW and HfMoNbTaW in the as-cast state and the as-homogenized state.

	State	Temperature (°C)				
		25	800	1200	1400	1600
Hf_0.5_MoNbTaW	As-cast	1465	790	790	695	545
	As-homogenized	1450	882	859	735	537
HfMoNbTaW	As-cast	1561	950	932	670	433
	As-homogenized	1569	974	954	840	571

**Figure 5.**
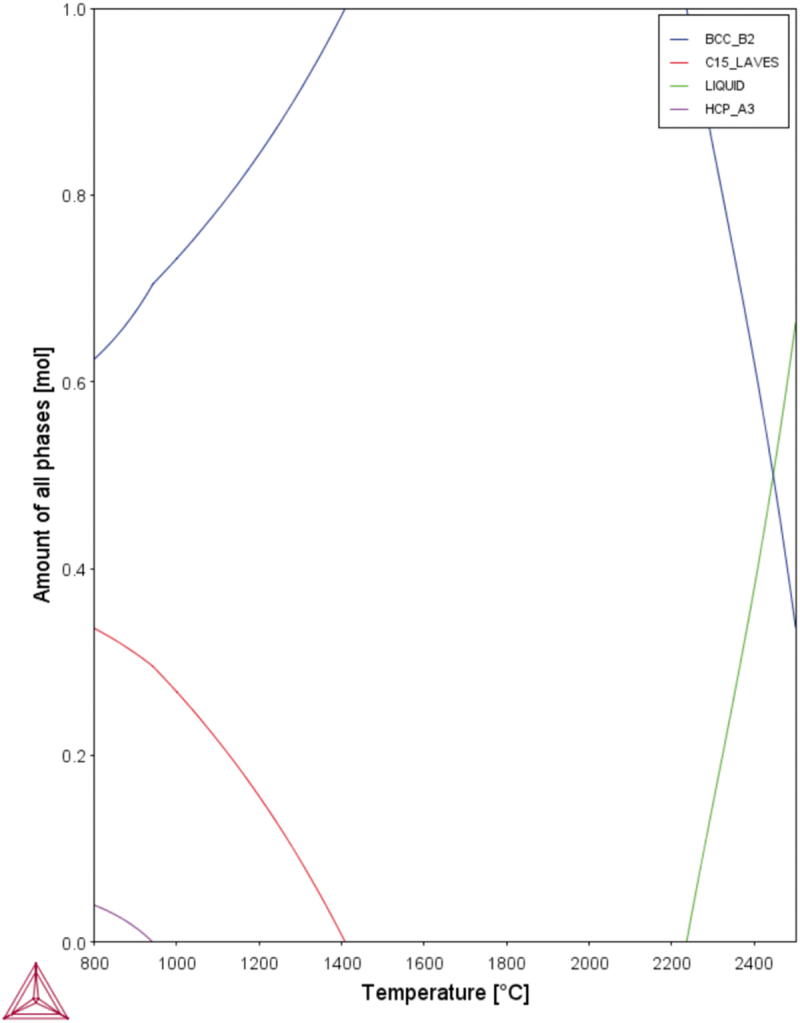
CALPHAD calculation result of HfMoNbTaW using the TCHEA4 thermodynamic database supplied by ThermoCalc.

Figure 4(e) shows the yield strengths of the experimental alloys at elevated temperatures, which are compared with those of MoNbTaW and MoNbTaVW [34] from room temperature to 1600°C. In the as-cast state, HfMoNbTaW possessed a higher elevated-temperature strength than MoNbTaVW below 1200°C, and Hf_0.5_MoNbTaW possessed a higher elevated-temperature strength than MoNbTaVW above 1200°C. The strength of HfMoNbTaW was even lower than those of Hf_0.5_MoNbTaW and MoNbTaVW at 1600°C. The CALPHAD calculation results are shown in Figure 5, for which the **TCHEA4** thermodynamic database supplied by ThermoCalc was employed. From Figure 5, solidification starts from 2480°C to 2220°C, which results in the dendritic structure. From Table 1, the interdendrite of HfMoNbTaW (D) is rich in 46% Hf, which causes the decrease of the solid solution-strengthening effect. The Hf-rich interdendritic phase possesses a lower melting point and weakens the solid-solution strengthening effect, so the strength at 1600°C is lower than MoNbTaVW.

This is also why Hf_0.5_MoNbTaW and HfMoNbTaW were homogenized at ultrahigh temperature of 2100°C for 2 h, which successfully allowed the Hf_0.5_MoNbTaW and HfMoNbTaW to form a single homogenous phase, respectively. From Figure 4(e), it can be seen that both the as-homogenized states of Hf_0.5_MoNbTaW and HfMoNbTaW revealed much higher strengths than MoNbTaW and MoNbTaVW, from room temperature to 1600°C. The as-homogenized state of HfMoNbTaW provided the highest strength at 1600°C, which was 571 MPa and became the highest of all the RHEAs without trace doping B or C.

The plateau strength shown in Figure 4(e) was present in the strength variation with temperature for each RHEA. For example, the plateau strength of HfMoNbTaW was approximately 950 MPa from 800 to 1200°C, while the plateau strength of MoNbTaVW was 550 MPa from 600 to 1000°C. We defined this temperature window as critical temperature *T*_*c*_, or knee temperature [36]. *T*_*c*_ is known to be an intrinsic property of body-centered cubic (bcc) materials, while the strengthening mechanism still remains unclear [37]. Below *Tc*, the strength of the material decreases when temperature increases or strain rate decreases. This softening phenomenon results from thermal activation of the slip of screw dislocation and the double-kink mechanism [38]. At *T*_*c*_ the strength of a material becomes independent of temperature or strain rate. Above *T*_*c*_ temperature, usually between 0.3 and 0.5 of the melting point, diffusion, grain boundary sliding, or dynamic recovery would occur, resulting in reduced strength as temperature increases again. One of the statements describing the plateau strength is that screw and edge dislocations have equal mobility at this *T*_*c*_ temperature [39]. Thus, plateau strength reflects a transition of slip mode from screw to edge dislocation during deformation.

## 4. Discussion

### 4.1 Strategy to optimize refractory alloy strength

High strength at ambient temperatures and high melting points of constituent elements are key factors that can contribute to the high-temperature strength of a refractory alloy at elevated temperatures. Table 3 shows the physical and mechanical properties of the refractory elements Cr, Hf, Mo, Nb, Ta, Ti, V, W, and Zr, in which Mo, Nb, Ta, and W have the highest melting points. These four elements were therefore selected for the preparations used in this study.

**Table 3.** Physical and mechanical properties of refractory elements [56].

Element	Cr	Hf	Mo	Nb	Ta	Ti	V	W	Zr
Melting point (K)	2130	2504	2896	2742	3293	1935	2163	3695	2128
Density (g/cm^3^)	7.2	13.3	10.2	8.6	16.7	4.5	6.1	19.3	6.5
Shear modulus (GPa)	115	30	125	38	69	44	46	161	33
Tensile strength (MPa)	103	240	438	240	170	195	310	550	280
Atomic radius (bcc) (Å)	1.24	1.55	1.36	1.43	1.43	1.42	1.32	1.37	1.57

The fifth element to be added to the MoNbTaW alloy must exhibit a strong interaction with the other four elements, and the increases in strength may be examined by using solid-solution-strengthening calculations. The solid-solution-strengthening mechanism of RHEAs was first proposed by Senkov et al. and then modified by Yao et al. [40,41]. The solution-strengthening value ∆*σ*_*i*_ contributed by element *i* is

(1) $$\Delta \sigma_{i}=AG{f_{i}}^{4/3}{c_{i}}^{2/3}$$

where *A* is a material-dependent dimensionless constant that equals 0.04, *G* represents the shear modulus of the alloy, *c*_*i*_ is the atomic fraction of element *i*, and *f*_*i*_ is the mismatch parameter of element *i*, which is related to its shear modulus and atomic size. *f*_*i*_ is calculated using the following expression:

(2) $$f_{i}=\sqrt{{\delta_{G,i}}^{2}+\alpha^{2}{\delta_{r,i}}^{2}}$$

where the value of *α* depends on the type of dislocation present [42]. For screw dislocation control, *α* is approximately 2–4. For edge dislocation control, *α* should not be lower than 16. For mixed dislocation, the *α* value is chosen to be 9. *δ*_*G,i*_ and *δ*_*r,i*_ are the modulus mismatch and atomic radius mismatch parameters, respectively, and represented by

(3) $$\delta_{G,i}=\frac{9}{8}\sum c_{j}\delta_{G,ij}$$

(4) $$\delta_{r,i}=\frac{9}{8}\sum c_{j}\delta_{r,ij}$$

There are nine atoms in the *i*-centered cluster in the bcc lattice and eight atoms neighboring the center atom, *i*. *δ*_*G,ij*_ and *δ*_*a,ij*_ are the differences in shear modulus and atomic radius between elements *i* and *j*, respectively, and represented by

(5) $$\delta_{G,ij}=\frac{2(G_{i}-G_{j})}{(G_{i}+G_{j})}$$

(6) $$\delta_{r,ij}=\frac{2(r_{i}-r_{j})}{(r_{i}+r_{j})}$$

where *G*_*i*_ and *G*_*j*_ are the shear moduli of elements *i* and *j*, respectively, and *r*_*i*_ and *r*_*j*_ are the atomic radiuses of elements *i* and *j*. The amount of solution strengthening ∆*σ* is the summation of all ∆*σ*_*i*_,

(7) $$\;\Delta \sigma \;=(\sum {\left(\Delta \sigma_{i}\right)}^{\;3/2}{\;)}^{\;2/3}$$

The calculated yield strength $\sigma_{y}^{cal}$ is the summation of ∆*σ* and the average yield stress $\sigma_{y}^{mix}$, according to the rule of mixing,

(8) $$\sigma_{y}^{mix}=\sum c_{i}\sigma_{i}$$

(9) $$\sigma_{y}^{cal}=\sigma_{y}^{mix}+\Delta \sigma$$

where *σ*_*i*_ is the yield strength of pure element *i*. Because the shear modulus of the HfMoNbTaW alloy system remains unknown, the average shear modulus of the alloy $G^{mix}$ is used to replace the shear modulus *G* to facilitate estimation. $G^{mix}$ is represented by

(10) $$G^{mix}=\sum c_{i}G_{i}$$

where *G*_*i*_ is the shear modulus of the pure element *i*. One can easily observe that higher *G* and *δ*_*G,i*_ or *δ*_*r,i*_ values would lead to an increase in the room-temperature strength of an RHEA. Furthermore, the melting point according to the rule of mixing $T^{mix}$ is defined by

(11) $$T^{mix}=\sum c_{i}T_{m,i}$$

where *T*_*m,i*_ is the melting point of pure element *i*. Elevated-temperature strength was estimated by assessing $\sigma_{y}^{cal}$ and $T^{mix}$. If the proposed RHEA possessed a higher $\sigma_{y}^{cal}$ and $T^{mix}$ than MoNbTaVW, a higher elevated-temperature strength than that of MoNbTaVW might be achieved. For illustrating the worse design, when Cr is added, the $\sigma_{y}^{cal}$ increases, but the $T^{mix}$ decreases, and the Laves phase might precipitate, which lessens solid-solution strengthening. When Ti is added, $\sigma_{y}^{cal}$ does not significantly change, but $T^{mix}$ decreases. When Zr is added, $\sigma_{y}^{cal}$ increases significantly, but $T^{mix}$ does not increase.

**Figure 6.**
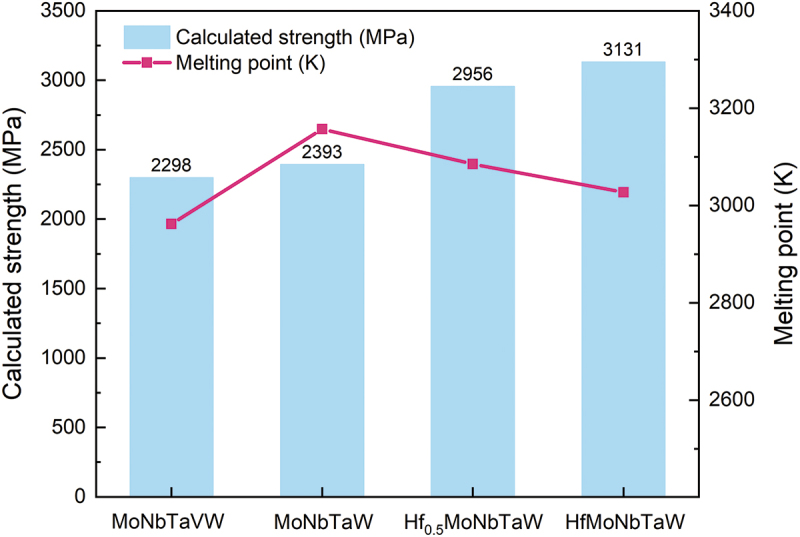
Theoretical yield strength $\sigma_{y}^{cal}$ and the rule-of-mixture melting point, $T^{mix}$ , of MoNbTaVW, MoNbTaW, Hf_0.5_MoNbTaW, and HfMoNbTaW calculated from equations (1) to (11) by assumptions *G* = $G^{mix}$ and *α* = 9.

In this study, Hf proved to be the optimal choice because it involved an increase in both $\sigma_{y}^{cal}$ and $T^{mix}$, since it possessed the lowest shear modulus and highest atomic radius among the Hf, Mo, Nb, Ta, and W elements and the highest melting point among the Cr, Hf, Ti, V, and Zr elements. Figure 6 shows the way in which the molar ratio of Hf affected the theoretical strength (v and melting temperature ($T^{mix}$) of MoNbTaW alloy systems. Although $T^{mix}$ decreased with the addition of Hf, $T^{mix}$ of equiatomic HfMoNbTaW was still higher than that of MoNbTaVW. Furthermore, the theoretical strength ($\sigma_{y}^{cal}$) increased 30.8% from 2393 to 3131 MPa during equiatomic addition of Hf into MoNbTaW and was thus significantly higher than that of MoNbTaVW. From the literature, we predict that the $\sigma_{y}^{cal}$ may be higher than the real strength of an alloy, because the $G^{mix}$ is referred to in our calculations, rather than the real shear modulus. The real shear modulus of MoNbTaVW is 75 GPa [43], which is lower than the $G^{mix}$ of 87.94 GPa. However, even if a value of 75 GPa was used in our calculations of $\sigma_{y}^{cal}$, the resulting strength is still approximately 2000 MPa, which is significantly higher than the experimental value of 1246 MPa [34]. The reasons for this result will be described in a later section.

### 4.2 The phase stability of HfMoNbTaW

Due to the extraordinary mechanical properties of the HfMoNbTaW alloy, we focus on this alloy for further discussion. From the binary phase diagram of Hf-Mo and Hf-W, HfMo_2_ and HfW_2_ form in the Laves phase with an AB_2_ structure [44]. However, the experimental alloys were designed using only solid-solution theoretical strengthening. Even in cases in which there is no other precipitate or cluster shown in the microstructure, long-term phase stability is still crucial at elevated temperatures. Phase formation empirical rules for HEAs from the literature were applied in the case of the experimental alloys, and the detailed calculation procedures for each parameter are shown below.

Thermodynamics parameters, atomic size parameter, and electronic parameters are used to examine the tendency of solid-solution formation [45,46]. The thermodynamics parameters are mixing entropy *∆S*_*mix*_, mixing enthalpy *∆H*_*mix*_, and *Ω* and represented as

(12) $$S_{mix}=-\sum c_{i}\;ln\;c_{i}$$

(13) $$\;\Delta H_{mix}\;=\sum \;4\;\Delta H_{ij}c_{i}c_{j}\;,i\neq \;j$$

(14) $$\;\Omega =\frac{{\;T}_{\;m}\;\Delta {\;S}_{\;mix}}{\left|\;\Delta H_{mix}\right|}$$

where *R* is the gas constant and ∆*H*_*ij*_ is the enthalpy of the binary liquid state of elements *i* and *j* at an equiatomic composition from Miedema’s model [47,48]. The atomic size parameter is atomic size difference *δ* represented as

(15) $$\delta \;=\;\sqrt{\sum c_{i}{\left(1-\frac{r_{i}}{\overset{ˉ}{r}}\right)}^{2}}$$

$\overset{ˉ}{r}\;$ is the average radius of the alloy defined by the rule of mixture:

(16) $$\overset{ˉ}{r}\;=\;\sum c_{i}r_{i}$$

Electronic parameters are the valence electron concentration *VEC* [49] and the Allen electronegativity difference Δ*χ*_*Allen*_ [50] represented as

(17) $$VEC=\sum c_{i}VEC_{i}$$

(18) $$\Delta X_{Allen}\;=\;\sqrt{c_{i}\left(1-\frac{X_{i}{\;}^{Allen}}{\overset{ˉ}{X}}\right)}$$

where *VEC*_*i*_ is the valence electron concentration of element *i* [51], $\chi_{i}^{Allen}$ is the electronegativity of element *i* from the study by Allen et al [52], and $\overset{ˉ}{X}\;$ is the average electronegativity of the alloy defined by the rule of mixture shown as

(19) $$\overset{ˉ}{X}\;=\;\sum c_{i}X_{i}^{Allen}$$

In Table 4, the parameters for each element are shown for the theoretical predictions. There are three criteria for predicting the phases and crystal structure of HEAs. The first rule is that a disordered solid-solution phase forms when *Ω* is higher than 1.1 and *δ* is below 6.6% [46]. This means that mixing enthalpy and atomic size difference are low enough not to form an ordered structure phase near the melting point. The second rule is that it is stable when rgw *VEC* is below 6.87 [49]. The third rule is that the Laves phase forms when ∆*χ*_*Allen*_ is higher than 7% and *δ* is higher than 5% in HEAs [53]. This is because the Laves phase forms when the electronegativity differences and atomic size differences between elements reach certain critical values. Table 4 shows the ∆*H*_*ij*_, $\chi_{i}^{Allen}$, and *VEC*_*i*_ for selected elements, while all the parameter calculation results for HfMoNbTaW are summarized in Table 5. The CALPHAD calculation results are also shown in Figure 5.

**Table 4.** The values of ∆*H*_*ij*_ (kJ/mol), $\chi_{i}^{Allen}$, and *VEC*_*i*_ of selected elements.

	Hf	Mo	Nb	Ta	W
Mo	−4	-	−6	−5	0
Nb	−4	−6	-	−5	0
Ta	3	−5	−5	-	−7
W	−6	0	0	−7	-
$\chi_{i}^{Allen}$	1.3	2.16	1.6	1.5	2.36
*VEC*_*i*_	4	6	5	5	6

**Table 5.** Summarized thermodynamics parameters, atomic size parameter, and electronic parameters of HfMoNbTaW.

	∆*H*_*mix*_ (kJ/mol)	∆*S*_*mix*_ (J/K∙mol)	*T*_*m*_ (K)	*Ω*	*δ* (%)	∆*χ*_*Allen*_ (%)	*VEC*
HfMoNbTaW	−5.44	1.6 R	3027	7.4	5.4	8.42	5.2

As shown in Table 5, the phase formation rules predict that HfMoNbTaW formed a disordered solid-solution phase, from the *VEC* and *Ω* values. The higher *Ω* value indicates that a disordered solid-solution phase not only formed near the melting point but also extended to the lower temperature region. However, from the ∆*χ*_*Allen*_ and *δ* values, the Laves phase was expected to precipitate in the cooling process during casting. Theoretically, HfMoNbTaW might begin to melt at approximately 2200°C and possess a stable disorder solid-solution phase from 1400°C to the melting point. The Laves phase would then precipitate below 1400°C, as shown in Figure 5. However, refractory elements usually exhibit slow diffusion processes. During solidification by casting, the water-cooled copper mold provided a higher cooling rate and thus inhibited Laves precipitation. After homogenization treatment, the air-cooling process led to a temperature drop from 2100 to 800°C in only 5 minutes and a drop to room temperature in 2 h. It was thus difficult for the Laves phase to form in such a short period of time, due to the low diffusion rate of RHEAs. SEM images, EDS mapping, and XRD patterns also prove the presence of a single phase after homogenization treatment. The solid-solution-strengthening effect may be discussed in detail only without elemental segregation and precipitates.

### 4.3 Correction of the theorical yield strength for plateau strength

A theoretically predicted $\sigma_{y}^{cal}$ value is generally larger than the experimental value. Here, we reviewed all the assumptions employed in our calculations and developed a new theory. First, the Young’s modulus of HfMoNbTaW was measured using nanoindentation techniques and was used to calculate the experimental shear modulus. This could be obtained using the following equation [54]:

(20) $$\;\;E\;\;=\;\frac{\;1-\nu^{\;2}}{\frac{\;1}{E_{r}}-\;\;\frac{\;1-{\nu_{i}}^{\;2}}{E_{i}}}$$

where *E* and *ν* are the Young’s modulus and the Poisson’s ratio of the test material, respectively, *E*_*r*_ is the reduced Young’s modulus of the test material measured by nanoindentation, *E*_*i*_ and *ν*_*i*_ are the Young’s modulus and the Poisson’s ratio of the diamond indenter, respectively, *ν* is equal to 0.35, which is the common value for RHEAs [43], and the *E*_*i*_ and *ν*_*i*_ values are 1141 GPa and 0.07, respectively. Thus, the *E* was calculated to be 188 ± 3 GPa. By using the expression: *G* = *E*/[2(1 + *ν*)], we further obtained a shear modulus of 73 ± 1 GPa for HfMoNbTaW.

However, the assumption of *α* = 9 that we used for mix dislocation control was an overestimate because deformation is usually controlled by screw dislocation at low temperatures under real conditions [40], which is represented by a value of *α* = 2. If one assumes values of G = 73 and *α* = 2 for the solid-solution-strengthening calculation, $\sigma_{y}^{cal}$ is equal to 2002 MPa. This value is much closer to the yield strength of 1569 MPa of HfMoNbTaW and close to the predicted strength of 1903 MPa estimated from the hardness value of 571 HV in the as-homogenized state. Edge dislocation theory has recently been applied to explain the phenomena of high strength at high temperatures and plateau strength in RHEAs [7,8]. This is because edge dislocation has been previously observed at a low-strain rate and at high temperatures. The scenario becomes that an assumption of *α* = 2 is reasonable as deformation is controlled by screw dislocations at lower temperatures, and thermal activation causes a reduction in strength as temperature increases up to *T*_*c*_ and the plateau strength in a range of temperatures is a transition of slip mode from screw dislocation to edge dislocation, which means that strength at high temperatures is progressively controlled by new slip mode of edge dislocations. Thus, we will attempt to explain the plateau strength by inference to check whether the edge dislocation theory can explain the origin of high strength at high temperatures or not.

**Figure 7.**
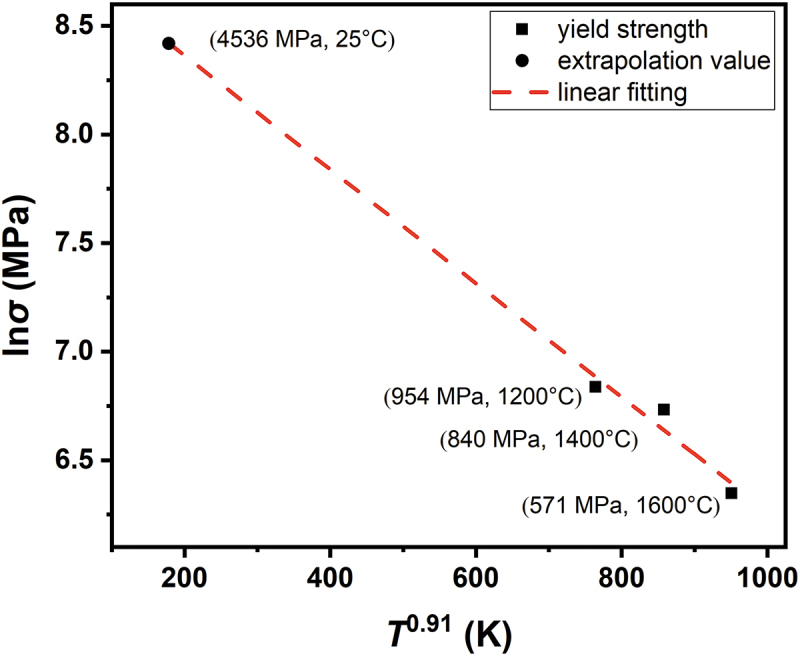
The plot of ln *σ* vs. *T*^0.91^ for *T* based on equation (22) and the data at 1200, 1400, and 1600°C. The linear fitting and extrapolation method were used to estimate the strength value at 25 °C.

First, strength at room temperature is predicted by the fitting equation from high-temperature strength so that we could see its consistency with that predicted from solid-solution-strengthening calculations under edge dislocation control. The relationship between strength, strain rate, and temperature has been established by Leyson et al. from the thermal activation Arrhenius model [55]. When the temperature increases, dislocation would move to other position easier, so the yield strength decreases. Therefore, no matter temperature is above or below T_c_, the reason for the decreasing yield strength when temperature elevates is thermal activation. Grain boundary sliding and dynamic recovery are all important factors, but we choose not to discuss here. This results in three equations in different finite temperature ranges. Maresca et al. used an ad hoc adjustment to accurately approximate them across the full temperature range using the following equation [8]:

(21) $$\sigma \text{=}\;\sigma_{\;\text{0}}\text{exp}-\frac{1}{0.55}\frac{kT}{\Delta E_{b}}\ln\;{\dot{\varepsilon }}_{0}\dot{\varepsilon }0.91$$

where *σ* is the yield stress, *σ*_*0*_ is the zero-temperature yield stress, *k* is the Boltzmann constant, ∆*E*_*b*_ is the characteristic energy barrier, $\dot{\varepsilon }$ is the strain rate, and ${\dot{\varepsilon }}_{0}$ is the reference strain rate. By applying a natural logarithm to both sides, this equation can be expressed as

(22) $$\text{ln}\;\sigma \text{=ln}\sigma_{\;\text{0}}\text{-}\frac{1}{0.55}\frac{k}{\Delta E_{b}}\text{ln}{\dot{\varepsilon }}_{0}\dot{\varepsilon }0.91T^{0.91}$$

with the form y = a + bx. Figure 7 shows a plot of ln *σ* vs. *T*^0.91^ for *T* = 1200, 1400, and 1600°C. After linear fitting, the extrapolation method was used to estimate strength at 298 K, which provided a value of 4536 MPa. Meanwhile, *α* = 16 was adopted for edge dislocation control in the equations from (1) to (9) for solid-solution strengthening, providing a value of $\sigma_{y}^{cal}$ = 4206 MPa. These two values calculated from different equations are highly consistent with each other and within a 7.2% difference, which indicates that the strength results from the dominant slip mode of edge dislocations above *T*_*c*_. This also demonstrates that the strength of the HfMoNbTaW alloy can only come from the activation of screw dislocations at room temperature, because the strength of the edge dislocation is too high. Therefore, the whole scenario is that the strength decreases with increasing temperature due to thermal activation of screw dislocations until *T*_*c*_. The alloy exhibits gradually increasing work-hardening capacity when temperature exceeds *T*_*c*_; as a result, the activated edge dislocation begins to contend in general yielding after microyielding along grain boundaries and results in plateau strength over a wide temperature range. The edge dislocation becomes dominant in general yielding and results in high strength at high temperatures above *T*_*c*_.

We also calculated for MoNbTaVW with α = 2 and α = 16, and the $\sigma_{y}^{cal}$ values were 1993 MPa and 2915 MPa. Then, we plot the ln*σ* of MoNbTaVW vs. *T*^0.91^ for *T* equals to 1200°C, 1400°C, and 1600°C. After linear fitting, the extrapolation method is used to estimate the strength at 298 K, which is 2950 MPa. Although 1993 MPa is not close to the 1246 MPa for room temperature strength of MoNbTaVW, 2915 MPa is almost consistent with 2950 MPa. Also, the temperature-strength curve of MoNbTaVW shows the plateau strength around 840 MPa and the T_c_ window around 600°C and 800°C, which means that there is a transition of slip mode. As a result, the room temperature strength of MoNbTaVW is controlled by screw dislocation, and the high temperature strength is controlled by edge dislocation.

We believe that this mechanism can comprehensively explain the *T*_*c*_, plateau strength phenomena and high elevated-temperature strength that occurs in bcc-type RHEAs.

## 5. Conclusions

1. Refractory Hf_0.5_MoNbTaW and HfMoNbTaW RHEAs with ultra-high temperature strength have been designed using high-melting-point elements and verified by the solid-solution strengthening theory.
2. After homogenization treatment at 2100°C for 2 h with subsequent air cooling, the RHEAs reveal no composition segregation and precipitates.
3. The HfMoNbTaW in the as-homogenized state provides the highest elevated-temperature yield strength of 571 MPa at 1600 °C.
4. The plateau strengths of the Hf_0.5_MoNbTaW and HfMoNbTaW alloys displayed in the medium range of elevated temperatures have been demonstrated to be resulted from the transition of slip mode from screw dislocation to edge dislocation.
5. The whole scenario of the evolution of strength with temperature is that the strength decreases with increasing temperature due to thermal activation of screw dislocations up to *T*_*c*_, the alloy exhibits gradually increased work-hardening capacity when temperature exceeds *T*_*c*_, the activated edge dislocation begins to contend in general yielding after microyielding along grain boundaries and results in plateau strength over a wide temperature range, and thus, the edge dislocation becomes dominant in general yielding and results in high strength at high temperatures above *T*_*c*_.
